# Examining the additive risk of TBI and comorbid conditions on dementia in military veterans: a retrospective cohort study

**DOI:** 10.1186/s13195-026-02086-5

**Published:** 2026-05-28

**Authors:** Alexandra L. Clark, Elizabeth Valocchi, Makenna B. McGill, Francesca V. Lopez, Catherine Chanfreau-Coffinier, Kelsey R. Thomas, Dylan J. Jester, Mark W. Logue, Victoria C. Merritt

**Affiliations:** 1 Research Service, VA San Diego Healthcare System, San Diego, CA USA; 2 Department of Psychology, The University of Texas at Austin, Austin, TX USA; 3 Department of Psychiatry, UC San Diego School of Medicine, La Jolla, CA USA; 4 Center of Excellence for Stress and Mental Health (CESAMH), VA San Diego Health Care System, San Diego, CA USA; 5 Center for the Study of Healthcare Innovation, Implementation and Policy (CSHIIP), VA Greater Los Angeles Health Care System, Los Angeles, CA USA; 6 War Related Illness and Injury Study Center (WRIISC), VA Palto Alto Health Care System, Palo Alto, CA USA; 7 National Center for PTSD, Behavioral Sciences Division, VA Boston Healthcare System, Boston, MA USA; 8 Department of Psychiatry, Boston University Chobanian and Avedisian School of Medicine, Boston, MA USA; 9 Biomedical Genetics, Boston University Chobanian and Avedisian School of Medicine, Boston, MA USA; 10 Department of Biostatistics, Boston University School of Public Health, Boston, MA USA

**Keywords:** Dementia, ADRD, Military Veterans, Traumatic brain injury, Mental health, VA Million Veteran Program

## Abstract

**Background:**

Although traumatic brain injury (TBI) has been identified as a risk factor for Alzheimer’s disease and related dementias (ADRD), not all studies have shown a clear link between TBI and ADRD, suggesting that the relationship between TBI and ADRD is complex, nuanced, and likely influenced by a multitude of factors. The purpose of this retrospective cohort study was to examine interactions between TBI history and co-occurring health conditions and health behaviors on 10-year incidence of ADRD among Veterans enrolled in the VA Million Veteran Program (MVP).

**Methods:**

Participants (*N* = 245,949) included Veterans aged ≥ 65 years at study enrollment who completed the MVP Baseline Survey and had VA electronic health record (EHR) data. Participants were followed from the date of MVP enrollment until the earliest ADRD diagnosis, death, or last visit date before the end of the observation period (January 2011 through September 2021). TBI status (TBI + vs. TBI-) and health conditions/health behaviors were characterized using a combination of MVP survey and EHR data. ADRD status (ADRD + vs. ADRD-) was based on a validated algorithm using EHR-extracted ICD codes. Cox proportional hazards regression analyses adjusted for age, sex, and education were used to assess the association between each health condition/health behavior and the hazard of ADRD in the TBI + and TBI- groups. Additive interactions between TBI and health conditions/health behaviors were tested using the relative excess risk due to interaction (RERI) statistic.

**Results:**

Among Veterans with a TBI history (*n* = 7,613), 12.16% developed ADRD over 10 years of follow-up; among those without TBI (*n* = 238,336), 4.32% developed ADRD. RERI analyses showed significant additive TBI by health condition/health behavior interactions for depression (RERI = 1.55, 95% CI = 0.96–2.15), heart attack/coronary artery disease (RERI = 0.76, 95% CI = 0.29–1.24), and physical inactivity (RERI = 0.58; 95% CI = 0.10–1.05), such that ADRD risk in Veterans with TBI was increased for those with these health conditions/health behaviors compared to those without.

**Conclusions:**

Findings suggest that ADRD risk following TBI may be heightened in the presence of certain health conditions/health behaviors, highlighting targeted areas of intervention for potentially mitigating adverse late-life outcomes in Veterans with a history of TBI.

**Supplementary Information:**

The online version contains supplementary material available at 10.1186/s13195-026-02086-5.

## Background

Given the increasing prevalence of Alzheimer’s disease (AD) and AD-related dementias (ADRD), and the associated costs of caring for people living with ADRD, there has been a great deal of work examining modifiable risk factors that may serve as important prevention and intervention targets for ADRD [1–3]. Traumatic brain injury (TBI) has been established as an important risk factor for ADRD [4, 5], with prior studies demonstrating approximately 1.5- to 2-fold increased risk of dementia following TBI [6–10]. However, the magnitude of this association is not uniform across studies, suggesting that the relationship between TBI and ADRD is complex and influenced by multiple factors. TBI severity is one important factor of influence in these differences [6, 11, 12], and the highly heterogeneous nature of TBI has likely further complicated efforts to detect consistent effects [13]. However, another possibility is that the long-term impact of TBI may depend upon other individual-level health and behavioral factors (e.g., cardiovascular disease, depression, alcohol use) that have not consistently been considered in existing TBI research studies [14–18].

Although several studies have established that the adverse effects of TBI are exacerbated in individuals with genetic vulnerability for AD [14, 19], research exploring the interactive effects of TBI and other modifiable health and behavioral conditions on ADRD remains limited [15–18]. This research gap is particularly important given nearly 45% of ADRD risk may be attributable to modifiable behavioral, lifestyle, and health factors [4, 5]. Recent critical reviews of the TBI literature have underscored the need for more nuanced considerations of co-occurring behavioral, lifestyle, and health factors in individuals with TBI [20–22]. Furthermore, the NIH-NINDS TBI Classification and Nomenclature Initiative recently introduced the Clinical, Biomarker, Imaging, and Modifier (CBI-M) Framework, which offers a more comprehensive approach for characterizing TBI [23, 24]. Notably, the “modifier” pillar of this framework emphasizes the importance of considering patient-specific factors—such as mental health history, medication use, and other systemic injuries—to enhance understanding of post-TBI outcomes and recovery trajectories [24]. However, the establishment of large-scale databases and longitudinal cohorts that integrate rich clinical and biological data is needed to ultimately advance this research.

The VA Million Veteran Program (MVP) offers a unique opportunity to study associations between TBI and other potentially modifiable risk factors on ADRD risk in a Veteran sample. MVP is a nationwide research initiative with the primary goal of improving the understanding of factors that influence health and wellness in Veterans [25]. To date, nearly half a million MVP participants are aged 65+, and the linking of comprehensive survey data with EHR data allows for more robust and complete characterization of modifiable risk factors. In the present study, we leveraged both MVP survey and EHR data to examine interactions between TBI and a wide range of risk factors—specifically health conditions and health behaviors—on 10-year incidence of ADRD. Our specific goal was to better understand the heterogeneous impact of TBI on dementia risk in Veterans. Doing so will allow us to build upon key recommendations and recent updates to TBI diagnostic frameworks to provide clarity regarding how co-occurring risk factors may ultimately shape long-term outcomes following TBI. We hypothesized that the combined effect of TBI and other health conditions/health behaviors (e.g., cardiovascular conditions, psychiatric conditions) would be associated with a greater risk of ADRD than either exposure alone.

## Methods

### VA Million Veteran Program (MVP)

Data from MVP, a national research program operated through the VA Office of Research & Development (ORD), were utilized in the present study. MVP was approved by the VA Central Institutional Review Board (cIRB) in 2010 and recruitment efforts began in 2011. MVP enrollment involves providing a blood sample for genetic analysis and consenting to researchers accessing their VA EHR, including care received both within and outside of the VA healthcare system (i.e., community care). Veterans are also asked to complete two comprehensive surveys at the time of MVP enrollment, referred to as the MVP Baseline Survey and MVP Lifestyle Survey [26], which were designed to complement information contained in the EHR. Surveys are self-administered and can be completed at home, either through hard copy or online (the online survey administration was introduced during the COVID-19 pandemic). The MVP Baseline Survey takes approximately 20 min to complete and assesses participant sociodemographic characteristics, medical history of self and family, and healthcare utilization. The MVP Lifestyle Survey takes approximately 45 min to complete and assesses daily lifestyle habits (such as physical activity, smoking, diet/nutrition), military and environmental exposures, and mental health and well-being. The surveys were developed by MVP and pilot work was completed prior to the study launch to evaluate tolerability of survey length, administration method (in-person vs. self-administered), and timing of survey completion (pre- vs. post-enrollment) [26]. Approximately 60% of MVP enrollees have completed the MVP Baseline Survey and 45% have completed the MVP Lifestyle Survey [26]. Research has demonstrated that the surveys can be used to independently examine Veteran health and behavioral outcomes to enhance our understanding of MVP enrollees [25, 26].

### Data sources and variable definitions

**Fig. 1:**
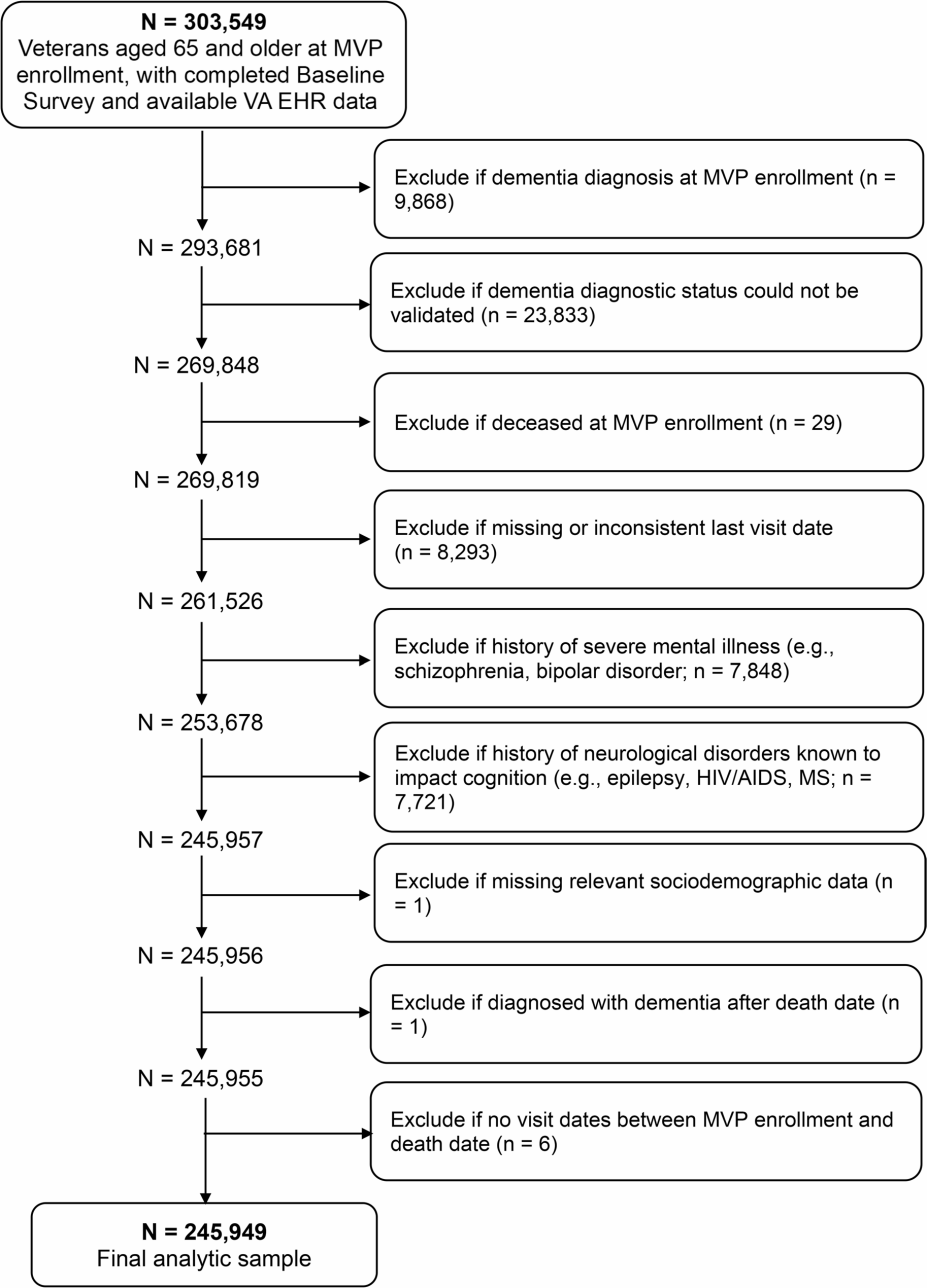
Flow chart depicting inclusion and exclusion criteria for final analytic sample of *N*=245,949. Abbreviations: MVP = Million Veteran Program; EHR = electronic health record; HIV = human immunodeficiency virus; AIDS = acquired immunodeficiency syndrome; MS = multiple sclerosis

This retrospective cohort study utilized data from the MVP 21.1 phenotype release, with a defined study period of January 1, 2011 through September 30, 2021. Veterans aged ≥ 65 years at MVP enrollment who completed the MVP Baseline Survey and had VA EHR data were eligible (*N* = 303,549). See Fig. 1 for a detailed flowchart of the study exclusion criteria. The final analytic sample included 245,949 Veterans. Participants were followed from the date of MVP enrollment until the earliest ADRD diagnosis, death, or last visit date before the end of the observation period, whichever occurred first. Administrative censoring occurred in 183,663 (74.68%) participants. Median follow-up was 4.97 years (interquartile range: [2.90–7.24]). Missing data on covariates were minimal (< 2%) and were handled using complete-case analysis. Sample sociodemographic characteristics (e.g., age at MVP enrollment, sex, education, race, ethnicity, income) were derived from the MVP Baseline Survey (details below) and supplemented with EHR data when necessary.

#### ADRD

VA EHR data were used to operationalize the primary outcome of interest and consisted of a validated ADRD case/control phenotype that has been detailed in prior work [27]. Specifically, ADRD status was defined using International Classification of Diseases (ICD) codes from the EHR. To be classified as having ADRD, a Veteran was required to have two or more ICD-9/10 codes for AD, non-specific dementia, or other related dementia, documented on different dates [14, 27]. See supplementary materials (sTable 1) for the complete list of ICD codes used to define ADRD. Furthermore, to be diagnosed with ADRD, age of onset (as defined by the first documented ADRD-related ICD code) was set to ≥ 65. Veterans without ADRD were defined as a Veteran aged 65 + at MVP enrollment who did not have *any* dementia-related ICD-9/10 codes, including ICD codes for mild cognitive impairment, and who had never been prescribed any AD-related medication.

#### TBI

TBI status was derived using a combination of data from the MVP Baseline Survey and ICD codes from the EHR [28]. This approach was used to maximize sensitivity for *lifetime* TBI ascertainment by incorporating evidence from any available TBI data source, rather than prioritizing one data source over another. Given that TBI events may occur outside of VA care or prior to EHR documentation, reliance on a single source may underestimate true exposure, and recent literature has demonstrated that combining TBI data sources in MVP (i.e., combining survey data with TBI ICD codes) produces a TBI phenotype with the strongest performance characteristics [28].

Within the MVP Baseline Survey, Veterans were presented a list of medical conditions and asked to report whether they have *ever* been diagnosed with any of the conditions; two items from the list pertain to TBI: (1) “traumatic brain injury” and (2) “concussion or loss of consciousness” (referred to as “BL-TBI” and “BL-CC”, respectively). Veterans were considered to have a history of TBI if they endorsed either the “BL-TBI” *or* “BL-CC” item on the survey. If neither “BL-TBI” nor “BL-CC” was endorsed, ICD codes from the EHR were evaluated. Specifically, ICD-9/10 codes associated with TBI were extracted from the EHR using a pre-determined list of codes generated by the Armed Forces Health Surveillance Branch of the Military Health System (see sTable 2). If one or more inpatient or outpatient ICD codes for TBI were present, the Veteran was considered to have a positive history of TBI. If no ICD codes for TBI were present, the Veteran was considered to have a negative history of TBI. Thus, a positive history of TBI (TBI+) was defined as endorsement of any one or more of the following variables: (1) BL-TBI, (2) BL-CC, or (3) at least one ICD-9/10 code for TBI, while a negative TBI history (TBI-) was defined as no evidence of TBI from all available TBI data sources.

#### Health conditions and health behaviors

This set of variables included a wide range of health conditions and health behaviors—all of which were derived from the MVP Baseline Survey and supplemented with EHR data when necessary. Health conditions included alcohol use disorder, congestive heart failure, depression, diabetes, hearing loss, heart attack/coronary artery disease (CAD), high blood pressure, high cholesterol, obesity, posttraumatic stress disorder (PTSD), pulmonary embolism/deep vein thrombosis (DVT), spinal cord injury, and vision loss. Health behaviors included physical activity, sleep, and smoking. See sTable 3 for more details on variable definitions and data sources.

### Data analysis

All analyses were performed with R version 3.5.0. The sample was divided into two groups: those with a history of TBI (i.e., TBI+) and those with no history of TBI (i.e., TBI-). Wilcoxon rank sum tests or chi-square tests were used to examine differences in sociodemographic variables, military characteristics, and health conditions and health behaviors between Veterans with and without ADRD within each of the TBI + and TBI- groups. Cox proportional hazards regression analyses were used to assess the association between each health condition/behavior and the hazard of ADRD in the TBI + and TBI- groups. All Cox proportional hazards models were adjusted for age at MVP enrollment, sex, and education. The proportional hazards assumption was assessed using Schoenfeld residuals. Adjusted hazard ratios (HR’s) and 95% confidence intervals (CI’s) were reported for each predictor variable. Given the large number of statistical comparisons, the Benjamini-Hochberg false discovery rate (FDR) correction was used to control for Type I error. The Benjamini-Hochberg method sorts *p*-values from each analysis and converts them to FDR-adjusted *p-*values, known as *q*-values, with *q* < 0.05 considered statistically significant.

We also tested for potential additive interactions of TBI and the set of health conditions and health behaviors by calculating the relative excess risk due to interaction (RERI) statistic, adjusting for age, sex, and education [29]. The RERI statistic quantifies whether the combined effect of two variables exceeds their main effects, suggesting an additive relationship. In other words, the RERI measures whether the combined risk associated with both variables exceeds the risk associated with each individual variable. Additive interaction was selected a priori given its relevance for identifying excess risk associated with joint exposures and its utility for informing prevention and intervention efforts. We computed 95% confidence intervals (CIs) for the RERI distribution using a 10,000-replicate bootstrap simulation. RERI estimates with 95% CIs that do not include 0 were considered statistically significant (i.e., if 95% CI all > 0 or < 0); a RERI value > 0 in indicates a combined effect that is more-than-additive (i.e., positive interaction), while a RERI value < 0 indicates a combined effect that is less-than-additive (i.e., a negative interaction) [29].

## Results

### Sample characteristics

**Fig. 2:**
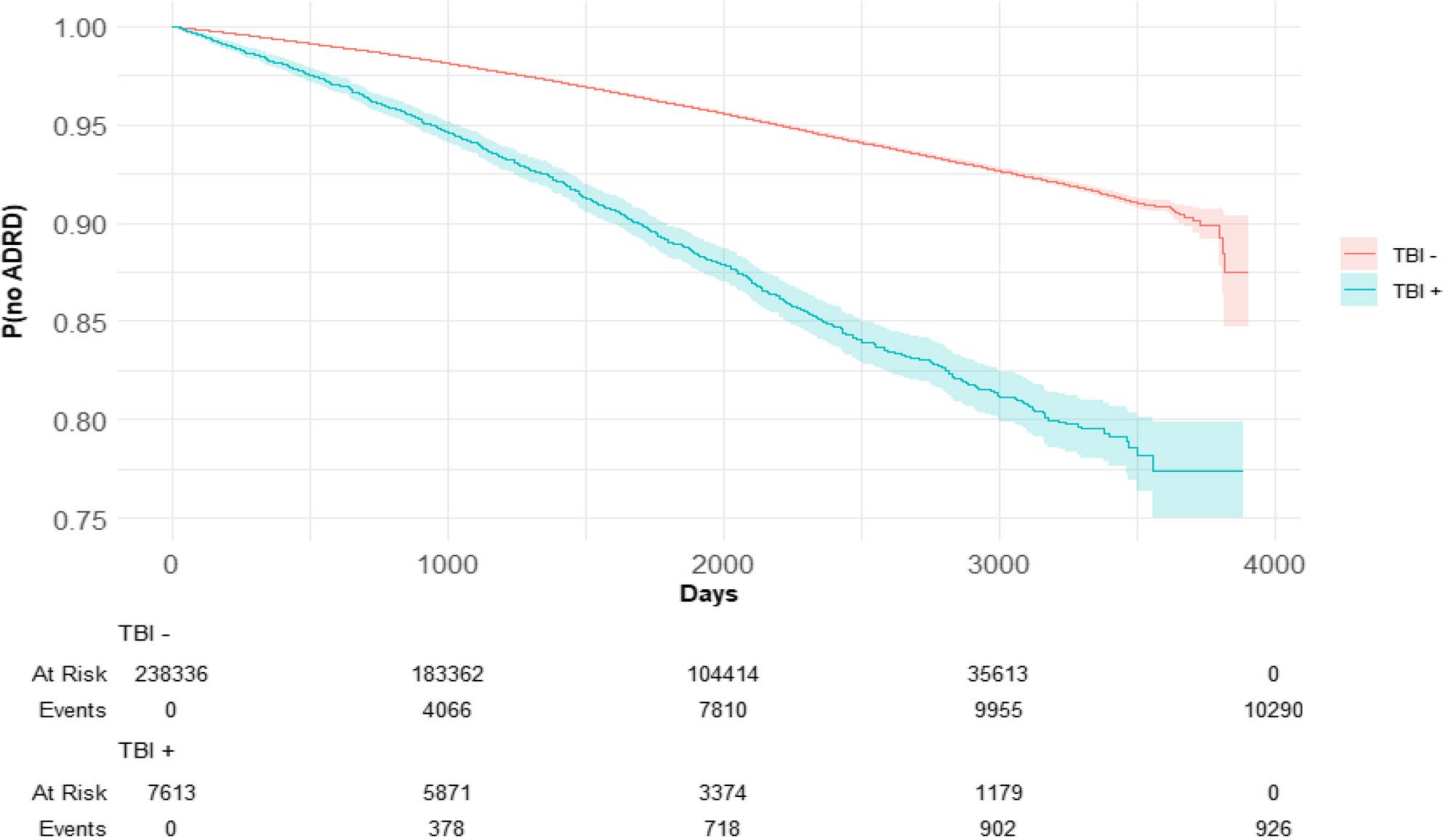
Kaplan-Meier curve illustrating the probability of remaining ADRD-free over 10 years of follow-up among Veterans with and without TBI. The y-axis reflects the estimated survival probability (P[no ADRD]) and the x-axis represents time (in days) from baseline (MVP enrollment). Shaded regions denote 95% confidence intervals. Survival probabilities were consistently lower in the TBI+ group. At-risk counts and event numbers are displayed below the plot. Abbreviations: ADRD = Alzheimer’s disease and related dementias; TBI = traumatic brain injury

Participant sample characteristics are detailed in Table 1. Of the 7,613 Veterans with a history of TBI, 12.16% (*n* = 926) developed ADRD over 10 years of follow-up. Of the 238,336 Veterans without a history of TBI, 4.32% (*n* = 10,290) developed ADRD over 10 years of follow-up. See Fig. 2 for a Kaplan-Meier curve depicting the probability of remaining disease-free over 10 years of follow-up. Among both the TBI + and TBI- groups, Veterans with ADRD were significantly older at MVP enrollment and had lower levels of education when compared to Veterans without ADRD. Additionally, Veterans with ADRD generally had higher rates of health conditions and poorer health behaviors compared to Veterans without ADRD in both TBI + and TBI- groups. See Table 1 for details.

**Table 1:** Sample characteristics

Variable^a^	TBI+				TBI-			
	Overall	ADRD-	ADRD+	*p* ^b^	Overall	ADRD-	ADRD+	*p* ^b^
	*N* = 7,613	*N* = 6,687	*N* = 926		*N* = 238,336	*N* = 228,046	*N* = 10,290	
Sociodemographic								
Enrollment Age (median, Q1, Q3)	70 (67, 76)	70 (67, 75)	75 (69, 82)	<0.001	71 (68, 77)	71 (68, 77)	77 (71, 83)	<0.001
Enrollment Age (mean, SD)	72.51 (6.89)	72.05 (6.62)	75.88 (7.82)	<0.001	73.18 (6.84)	72.99 (6.75)	77.20 (7.53)	<0.001
Sex				0.206				0.810
Male	7,372 (96.83)	6,469 (96.74)	903 (97.52)		232,216 (97.43)	222,194 (97.43)	10,022 (97.40)	
Female	241 (3.17)	218 (3.26)	23 (2.48)		6,120 (2.57)	5,852 (2.57)	268 (2.60)	
Education				<0.001				<0.001
Advanced Degree	956 (12.81)	844 (12.88)	112 (12.32)		31,448 (13.41)	30,267 (13.48)	1,181 (11.74)	
College	4,462 (59.79)	3,989 (60.86)	473 (52.04)		136,316 (58.12)	131,021 (58.37)	5,295 (52.63)	
High School or Less	2,045 (27.40)	1,721 (26.26)	324 (35.64)		66,780 (28.47)	63,196 (28.15)	3,584 (35.63)	
Race				0.048				<0.001
White	6,231 (81.85)	5,475 (81.88)	756 (81.64)		201,473 (84.53)	193,121 (84.69)	8,352 (81.17)	
Black / African American	538 (7.07)	453 (6.77)	85 (9.18)		18,466 (7.75)	17,341 (7.60)	1,125 (10.93)	
American Indian	86 (1.13)	81 (1.21)	5 (0.54)		1,380 (0.58)	1,326 (0.58)	54 (0.52)	
Asian or Pacific Islander	52 (0.68)	46 (0.69)	6 (0.65)		1,891 (0.79)	1,827 (0.80)	64 (0.62)	
Multiracial	360 (4.73)	326 (4.88)	34 (3.67)		6,411 (2.69)	6,124 (2.69)	287 (2.79)	
Unknown	193 (2.54)	172 (2.57)	21 (2.27)		5,506 (2.31)	5,234 (2.30)	272 (2.64)	
Other	153 (2.01)	134 (2.00)	19 (2.05)		3,209 (1.35)	3,073 (1.35)	136 (1.32)	
Ethnicity				0.218				<0.001
Non-Hispanic/Non-Latino	7,118 (93.50)	6,264 (93.67)	854 (92.22)		224,804 (94.32)	215,223 (94.38)	9,581 (93.11)	
Hispanic/Latino	470 (6.17)	402 (6.01)	68 (7.34)		12,694 (5.33)	12,023 (5.27)	671 (6.52)	
Unknown	25 (0.33)	21 (0.31)	4 (0.43)		838 (0.35)	800 (0.35)	38 (0.37)	
Income				<0.001				<0.001
< $60,000	5,065 (75.78)	4,366 (74.45)	699 (85.24)		150,064 (71.65)	142,740 (71.18)	7,324 (82.13)	
$60,000 - $99,999	1,186 (17.74)	1,099 (18.74)	87 (10.61)		40,735 (19.45)	39,523 (19.71)	1,212 (13.59)	
>= $100,000	433 (6.48)	399 (6.80)	34 (4.15)		18,649 (8.90)	18,267 (9.11)	382 (4.28)	
Health Conditions								
Alcohol Use Disorder	840 (11.11)	730 (11.00)	110 (11.88)	0.427	10,291 (4.42)	9,576 (4.30)	715 (6.98)	<0.001
Congestive Heart Failure	2,014 (26.45)	1,673 (25.02)	341 (36.83)	<0.001	42,654 (17.90)	39,420 (17.29)	3,234 (31.43)	<0.001
Depression	4,107 (53.95)	3,515 (52.56)	592 (63.93)	<0.001	70,827 (29.72)	65,936 (28.91)	4,891 (47.53)	<0.001
Diabetes	3,737 (49.09)	3,246 (48.54)	491 (53.02)	0.011	106,571 (44.71)	101,316 (44.43)	5,255 (51.07)	<0.001
Hearing Loss	5,128 (67.36)	4,431 (66.26)	697 (75.27)	<0.001	143,873 (60.37)	136,716 (59.95)	7,157 (69.55)	<0.001
Heart Attack/CAD	3,693 (48.51)	3,131 (46.82)	562 (60.69)	<0.001	96,396 (40.45)	90,765 (39.80)	5,631 (54.72)	<0.001
High Blood Pressure	6,764 (88.85)	5,913 (88.43)	851 (91.90)	0.002	204,250 (85.70)	194,812 (85.43)	9,438 (91.72)	<0.001
High Cholesterol	6,650 (87.35)	5,819 (87.02)	831 (89.74)	0.020	203,684 (85.46)	194,526 (85.30)	9,158 (89.00)	<0.001
Obesity	4,150 (55.91)	3,686 (56.43)	464 (52.08)	0.014	115,613 (49.76)	110,776 (49.80)	4,837 (48.85)	0.064
PTSD	2,871 (37.71)	2,578 (38.55)	293 (31.64)	<0.001	42,215 (17.71)	40,155 (17.61)	2,060 (20.02)	<0.001
Pulmonary Embolism/DVT	988 (12.98)	834 (12.47)	154 (16.63)	<0.001	19,038 (7.99)	17,770 (7.79)	1,268 (12.32)	<0.001
Spinal Cord Injury	928 (12.19)	827 (12.37)	101 (10.91)	0.203	13,236 (5.55)	12,553 (5.50)	683 (6.64)	<0.001
Vision Loss	735 (9.65)	605 (9.05)	130 (14.04)	<0.001	13,053 (5.48)	11,855 (5.20)	1,198 (11.64)	<0.001
Health Behaviors								
Physical Activity				<0.001				<0.001
Regular Exercise	3,029 (40.66)	2,699 (41.19)	330 (36.79)		101,509 (43.34)	97,711 (43.58)	3,798 (38.02)	
Less Frequent Exercise	1,763 (23.66)	1,579 (24.10)	184 (20.51)		57,901 (24.72)	55,653 (24.82)	2,248 (22.50)	
Never	2,658 (35.68)	2,275 (34.72)	383 (42.70)		74,807 (31.94)	70,864 (31.60)	3,943 (39.47)	
Sleep				0.008				<0.001
None	1,028 (19.48)	898 (19.32)	130 (20.63)		47,342 (26.51)	45,459 (26.55)	1,883 (25.63)	
Insomnia	1,872 (35.47)	1,632 (35.11)	240 (38.10)		68,342 (38.27)	65,481 (38.24)	2,861 (38.94)	
Poor Sleep Quality	628 (11.90)	540 (11.62)	88 (13.97)		17,935 (10.04)	17,059 (9.96)	876 (11.92)	
Both	1,750 (33.16)	1,578 (33.95)	172 (27.30)		44,969 (25.18)	43,241 (25.25)	1,728 (23.52)	
Smoking				0.162				0.019
Never	5,709 (76.09)	5,041 (76.34)	668 (74.22)		57,136 (24.25)	54,584 (24.21)	2,552 (25.23)	
Current/Former Smoker	1,794 (23.91)	1,562 (23.66)	232 (25.78)		178,445 (75.75)	170,883 (75.79)	7,562 (74.77)	

Actual n for each variable may be less due to missing data. Percentages are calculated based on the available n for each variable

*Abbreviations*: *TBI* Traumatic brain injury, *ADRD* Alzheimer’s disease and related dementias, *CAD *Coronary artery disease, *PTSD* Posttraumatic stress disorder, *DVT* Deep vein thrombosis

^a^n(%) unless otherwise specified

^b^Wilcoxon rank sum; Pearson's chi-square

### Main effects of health conditions and health behaviors on ADRD risk, stratified by TBI status

The results from the Cox proportional hazard regression analyses examining associations between health conditions and health behaviors on incident ADRD, stratified by TBI status, are reported in Table 2. Of all the health conditions evaluated, alcohol use disorder, congestive heart failure, depression, diabetes, heart attack/coronary artery disease, high blood pressure, high cholesterol, and pulmonary embolism/DVT were associated with increased risk for ADRD among Veterans with a history of TBI (hazards ratios [HR’s] = 1.19–2.10). Additionally, among Veterans with a TBI history, those who endorsed never exercising were at increased risk for ADRD (HR = 1.35). Among Veterans without a history of TBI, all health conditions were associated with an increased risk for ADRD (HR’s = 1.09–2.88). Furthermore, among Veterans without TBI, those who endorsed never exercising were at increased risk for ADRD (HR = 1.20), as were Veterans who endorsed poor sleep quality (HR = 1.26) and insomnia/poor sleep quality (HR = 1.08). See Table 2 for details.

**Table 2:** Associations of health conditions and health behaviors with ADRD in Veterans with and without TBI

Health Conditions^a^	TBI+				TBI-			
	*N*	HR	95% CI	*p*	*N*	HR	95% CI	*p*
Alcohol Use Disorder	7,410	1.44	1.17, 1.76	0.001	234,544	2.34	2.17, 2.53	<0.001
Congestive Heart Failure	7,463	1.43	1.24, 1.64	<0.001	234,544	1.88	1.80, 1.96	<0.001
Depression	7,463	2.10	1.83, 2.42	<0.001	234,544	2.88	2.76, 2.99	<0.001
Diabetes	7,463	1.19	1.05, 1.36	0.017	234,544	1.43	1.37, 1.49	<0.001
Hearing Loss	7,463	1.11	0.95, 1.29	0.248	234,644	1.09	1.05, 1.14	<0.001
Heart Attack/CAD	7,463	1.46	1.27, 1.67	<0.001	234,544	1.53	1.47, 1.59	<0.001
High Blood Pressure	7,463	1.22	0.96, 1.55	0.026	234,544	1.59	1.48, 1.71	<0.001
High Cholesterol	7,463	1.24	1.00, 1.54	0.087	234,544	1.36	1.27, 1.44	<0.001
Obesity	7,285	1.01	0.88, 1.33	0.987	228,839	1.24	1.19, 1.29	<0.001
PTSD	7,463	1.07	0.92, 1.24	0.479	234,544	1.79	1.70, 1.88	<0.001
Pulmonary Embolism/DVT	7,463	1.35	1.14, 1.61	0.001	234,544	1.60	1.51, 1.70	<0.001
Spinal Cord Injury	7,463	1.00	0.81, 1.22	0.987	234,544	1.29	1.19, 1.39	<0.001
Vision Loss	7,463	1.15	0.95, 1.39	0.217	234,544	1.52	1.43, 1.62	<0.001

Health Behaviors	*N*	HR	95% CI	*p*	*N*	HR	95% CI	*p*
Physical Activity	7,323				230,862			
Regular Exercise		--	--	--		--	--	--
Less Frequent Exercise		1.02	0.85, 1.23	0.861		1.05	0.77, 1.43	0.001
Never		1.35	1.17, 1.57	<0.001		1.20	0.93, 1.56	<0.001
Sleep	5,184				176,025			
None		--	--	--		--	--	--
Insomnia		1.06	1.00, 1.12	0.793		1.06	1.00, 1.12	0.109
Poor Sleep Quality		1.37	1.26, 1.49	0.189		1.37	1.26, 1.49	<0.001
Both		1.16	1.08, 1.24	0.796		1.16	1.08, 1.24	<0.001
Smoking	7,366				232,065			
Never		--	--	--		--	--	--
Current/Former Smoker		0.94	0.81, 1.09	0.509		0.96	0.92, 1.01	0.168

All models are adjusted for age, sex, and education. Reported *p*-values reflect FDR-corrected *p*-values

*Abbreviations*: *ADRD* Alzheimer’s disease and related dementias, *TBI* Traumatic brain injury, *CAD* Coronary artery disease, *PTSD* Posttraumatic stress disorder, *DVT* Deep vein thrombosis

^a^Reference is “No”

### Additive interactions: TBI x health conditions and health behaviors on ADRD

**Fig. 3:**
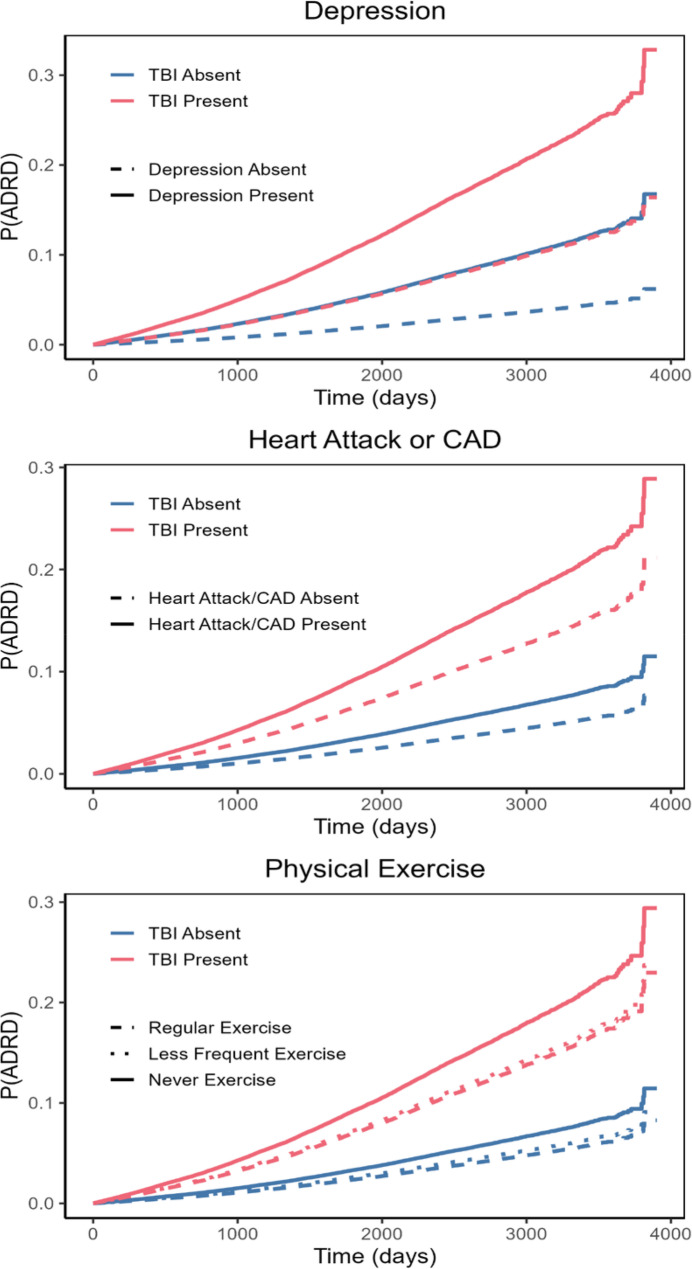
RERI plots depicting significant additive-scale interaction estimates with TBI and health conditions/behaviors on ADRD. The y-axis shows the estimated risk of ADRD as a function of time, TBI, and the health condition/behavior in MVP participants. The red lines represent the TBI+ group, while the blue lines represent the TBI- group. The solid lines represents the presence of the health condition/behavior, while the dotted lines represents the absence of the health condition/behavior. The distance between the lines (red solid vs. red dashed; blue solid vs. blue dashed) illustrates the greater ADRD risk associated with the presence of the health condition/behavior in Veterans with TBI relative to those without TBI. Abbreviations: RERI = relative excess risk due to interaction; ADRD = Alzheimer’s disease and related dementias; TBI = traumatic brain injury; CAD = coronary artery disease

Results of the RERI analyses adjusting for age, sex, and education are listed in Table 3. RERI analyses showed significant positive interactions of TBI and depression (RERI = 1.55, 95% CI: 0.96, 2.15), heart attack/CAD (RERI = 0.76, 95% CI: 0.29, 1.24), and physical inactivity (RERI = 0.58, 95% CI: 0.10, 1.05). In particular, ADRD risk was highest among Veterans with both TBI and these health conditions/behaviors, compared to those with either exposure alone. Figure 3 illustrates these additive interactions, highlighting the relationship between TBI and each health condition on ADRD risk as a function of time. For instance, at the end of the study, the difference in the estimated prevalence of ADRD was greater between those with versus without depression in the TBI+ group (16.43%) relative to the TBI- group (10.56%). Furthermore, the difference in the estimated prevalence of ADRD was greater between those with versus without heart attack/CAD in the TBI+ group (7.73%) relative to the TBI- group (3.81%). Finally, the estimated prevalence of ADRD was greater between those physically inactive versus active in the TBI+ group (6.43%) relative to the TBI- group (3.17%). In other words, Veterans with both TBI and these health conditions/behaviors were at greatest risk of developing ADRD.

**Table 3:** Additive-scale interaction results: TBI x health conditions and health behaviors on ADRD

	RERI (95% CI)
Health Conditions^a^	
Alcohol Use Disorder	0.05 (-0.92, 1.01)
Congestive Heart Failure	0.30 (-0.23, 0.83)
Depression	**1.55 (0.96**,** 2.15)**
Diabetes	0.26 (-0.20, 0.71)
Hearing Loss	0.11 (-0.35, 0.57)
Heart Attack/CAD	**0.76 (0.29**,** 1.24)**
High Blood Pressure	0.22 (-0.70, 1.13)
High Cholesterol	0.44 (-0.26, 1.15)
Obesity	-0.04 (-0.47, 0.39)
PTSD	-0.20 (-0.69, 0.30)
Pulmonary Embolism/DVT	0.41 (-0.26, 1.08)
Spinal Cord Injury	-0.24 (-0.87, 0.40)
Vision Loss	-0.25 (-0.88, 0.37)
Health Behaviors	
Physical Activity	
Regular Exercise	Reference
Less Frequent Exercise	-0.24 (-0.69, 0.22)
Never	**0.58 (0.10**,** 1.05)**
Sleep	
None	Reference
Insomnia	0.06 (-0.42, 0.54)
Poor Sleep Quality	0.37 (-0.45, 1.18)
Both	-0.26 (-0.79, 0.27)
Smoking	
Never	Reference
Current/Former Smoker	-0.07 (-0.51, 0.37)

Additive-scale interaction with TBI, estimated using the relative excess risk due to interaction (RERI) statistic. Bolded values imply additive interaction

*Abbreviations*: *TBI* Traumatic brain injury, *ADRD* Alzheimer’s disease and related dementias, *CAD* Coronary artery disease, *PTSD* Posttraumatic stress disorder, *DVT* Deep vein thrombosis

^a^Reference is “No”

## Discussion

The present study leveraged data from MVP to examine the association between TBI, health conditions/behaviors, and 10-year incidence of ADRD in MVP-enrolled Veterans. We utilized a combination of data from MVP surveys and the VA EHR to robustly characterize exposure variables of interest within a large, national dataset of Veterans, and examined potentially modifiable risk factors that have not yet been comprehensively examined in tandem in most epidemiological studies of TBI and ADRD. Among Veterans with a TBI history, roughly 12.16% developed ADRD over 10 years of follow-up; comparatively, only 4.32% of Veterans without TBI developed ADRD over the same period. The incidence of ADRD observed in this Veteran cohort appears comparable to or modestly higher than rates reported in community-based samples [30–32], which may reflect the greater burden of comorbidities and risk exposures in Veterans [15, 33]. Furthermore, when accounting for age, sex, and education, many of the health conditions evaluated were associated with an increased risk for ADRD in both TBI + and TBI- Veterans. Moreover, results revealed that the co-occurrence of TBI and certain health conditions and behaviors (i.e., depression, heart attack/CAD, physical inactivity) amplified the risk for ADRD, highlighting that the combination of neurologic injury and specific health factors may increase susceptibility for cognitive decline in late life. Findings reinforce key concepts from novel TBI diagnostic frameworks [24], which emphasize the need for more comprehensive examinations of co-occurring risk factors to better understand long-term outcomes following TBI.

The finding that incident ADRD cases exhibited higher rates of chronic health conditions and adverse health behaviors compared to their respective ADRD control groups in both TBI + and TBI- Veterans suggests that these health and behavioral factors likely play an important role in the development of ADRD and may contribute to ADRD risk independent of TBI history [16, 18, 34, 35]. Nevertheless, results also showed that there were more-than-additive effects of TBI and certain health conditions/behaviors (i.e., depression, heart attack/CAD, physical inactivity). While it has been well-established that addressing health and behavioral risk factors are important for reducing late-life risk for ADRD, our findings highlight the importance of consistent clinical management and treatment of co-occurring conditions and a sedentary lifestyle, especially within the context of a positive TBI history. Our results align with data from previous studies demonstrating significant interactions of TBI and depression [15] and TBI and cardiovascular disease [15, 18], although they contradict findings from another study that failed to find synergistic effects [16].

From a mechanistic perspective, TBI has been associated with prolonged inflammatory responses [36], and chronic inflammation is a known risk factor for both depression and ADRD [37, 38]. Additionally, there is growing recognition that TBI may disrupt cardiovascular and cerebrovascular functioning, impairing cerebral blood flow and increasing the risk for vascular comorbidities—both of which have been implicated in the pathogenesis of ADRD [39–43]. Similarly, not engaging in physical activity may worsen cardiovascular functioning and increase risk for dementia [44, 45]. It is possible that TBI directly contributes to the development of these health conditions and ultimately makes engagement in positive health behaviors that mitigate risk for ADRD difficult. However, it is equally plausible that risk factors develop independently of TBI and increase susceptibility to ADRD. While we cannot speak to the temporal ordering or development of these health conditions in relation to TBI, it is important to reiterate that health conditions and adverse health behaviors were generally higher in Veterans with ADRD compared to those without ADRD—both among TBI + and TBI- groups. Ultimately, prospective longitudinal cohort studies are needed for clarification, and close monitoring of biomarkers following TBI and over time may prove helpful. The increasing availability of blood-based biomarkers of inflammation, neurodegeneration, and AD-pathology also offers a promising opportunity to further elucidate the biological mechanisms linking TBI, co-occurring health conditions, and ADRD.

The strengths and limitations of this study should be considered when interpreting the findings. In addition to evaluating a large, nationwide cohort of Veterans, a notable strength of this study was the leveraging of both survey and EHR data to allow for robust characterization of TBI, other risk factors, and 10-year incidence of ADRD. The use of these complementary data sources helped reduce potential measurement bias by enabling a more complete ascertainment of exposures than would be captured with a single data source alone. We also used a validated ADRD phenotype as our outcome measure that reliably captures the range of dementia cases seen within the VA [27]. The use of this validated algorithm helped minimize ADRD misclassification inherent in diagnostic coding. Finally, the employment of RERI analytic techniques more appropriately estimated the public health impact of TBI in combination with other health factors/behaviors of interest, further reinforcing the practical implications of our findings.

Study limitations included the assessment of health conditions and health behaviors at a single point in time—i.e., at the time of MVP enrollment. Moreover, ICD codes do not adequately provide information about the severity of chronic medical conditions and whether these comorbidities were effectively managed by the patient and their physician(s); this will need to be considered in future research. We also note that while we utilized multiple data sources to assess TBI history, consistent with prior MVP TBI research recommendations [28], there are limitations associated with retrospective identification of TBI in the EHR (i.e., self-reported TBI via surveys may be subject to recall bias and ICD-based ascertainment of TBI from the EHR may not capture injuries that occurred outside of VA care). TBI severity was also not examined due to the TBI definition used in this study, and we were unable to incorporate total number of lifetime TBI’s, which will be an important variable to consider in the future. Other limitations relate to generalizability; given that our sample was predominantly male Veterans, findings may not generalize to female Veterans or to the broader civilian population. Finally, as with all observational studies, residual misclassification and unmeasured confounding remain possible and should be considered when interpreting these findings.

## Conclusions

The present study leveraged EHR and survey data from MVP to enhance our understanding of ADRD risk among Veterans. Findings underscore that TBI and certain health factors interact to further increase ADRD risk and clarify the importance of considering a wide range of co-occurring health conditions/behaviors along with TBI when assessing dementia risk. These results provide support for the “modifier” pillar of the NIH-NINDS TBI CBI-M model by demonstrating that patient specific health and lifestyle factors in the context of TBI history may play an important role in long-term dementia risk. These results also suggest that care for individuals with TBI should extend beyond acute injury management to include ongoing assessment and treatment of modifiable risk factors known to influence cognitive functioning and neural health. Moreover, increasing provider awareness of the interaction between TBI and specific risk factors (e.g., depression, cardiovascular disease, and physical inactivity) may facilitate earlier identification of “at-risk” patients and help improve screening and intervention initiatives for longitudinal follow-up care after a TBI. Future research will be needed to examine whether sustained management of these modifiable factors can alter ADRD trajectories among individuals with TBI.

## Supplementary Information

Supplementary Material 1.

## Data Availability

The data and code used to generate MVP results are accessible to researchers with MVP data access. Due to VA policy, MVP is currently only accessible to researchers with a funded MVP project (e.g., VA Merit Award, Career Development Award, NIH R01). Thus, the datasets generated and/or analyzed during the current study are not publicly available, but the corresponding author is willing to engage with reasonable requests, share code, and answer questions about the present study. Information about accessing MVP data can be found at:https://www.mvp.va.gov/pwa/joinmvp.

## Abbreviations

AD: Alzheimer’s disease
ADRD: Alzheimer’s disease and related dementias
ADRD +: Positive Alzheimer’s disease and related dementias history
ADRD-: Negative Alzheimer’s disease and related dementias history
AIDS: Acquired immunodeficiency syndrome
BL-CC: Baseline Survey concussion or loss of consciousness
BL-TBI: Baseline Survey traumatic brain injury
CAD: Coronary artery disease
CBI-M: Clinical, Biomarker, Imaging, and Modifier
CI: Confidence interval
CIRB: Central Institutional Review Board
DVT: Deep vein thrombosis
EHR: Electronic health record
FDR: False discovery rate
HIV: Human immunodeficiency virus
HR: Hazard ratio
ICD: International Classification of Diseases
MS: Multiple sclerosis
MVP: Million Veteran Program
NIH-NINDS: National Institutes of Health - National Institute of Neurological Disorders and Stroke
ORD: Office of Research and Development
PTSD: Posttraumatic stress disorder
Q1: First quartile
Q3: Third quartile
RERI: Relative excess risk due to interaction
SD: Standard deviation
TBI: Traumatic brain injury
TBI +: Positive TBI history
TBI-: Negative TBI history
VA: Veterans Affairs
